# Adipose Stem Cell-Derived Extracellular Vesicles Induce Proliferation of Schwann Cells via Internalization

**DOI:** 10.3390/cells9010163

**Published:** 2020-01-09

**Authors:** Maximilian Haertinger, Tamara Weiss, Anda Mann, Annette Tabi, Victoria Brandel, Christine Radtke

**Affiliations:** Research Laboratory of the Division of Plastic and Reconstructive Surgery, Department of Surgery, Medical University of Vienna, 1090 Vienna, Austria; maximilian.haertinger@gmail.com (M.H.); anda.mann@meduniwien.ac.at (A.M.); tannette79@hotmail.com (A.T.); victoria.brandel@gmx.net (V.B.); christine.radtke@meduniwien.ac.at (C.R.)

**Keywords:** Schwann cells, adipose-derived stem cells, extracellular vesicles, endocytosis, proliferation, nerve regeneration, nerve injury

## Abstract

Recent studies showed a beneficial effect of adipose stem cell-derived extracellular vesicles (ADSC-EVs) on sciatic nerve repair, presumably through Schwann cell (SC) modulation. However, it has not yet been elucidated whether ADSC-EVs exert this supportive effect on SCs by extracellular receptor binding, fusion to the SC membrane, or endocytosis mediated internalization. ADSCs, ADSC-EVs, and SCs were isolated from rats and characterized according to associated marker expression and properties. The proliferation rate of SCs in response to ADSC-EVs was determined using a multicolor immunofluorescence staining panel followed by automated image analysis. SCs treated with ADSC-EVs and silica beads were further investigated by 3-D high resolution confocal microscopy and live cell imaging. Our findings demonstrated that ADSC-EVs significantly enhanced the proliferation of SCs in a time- and dose-dependent manner. 3-D image analysis revealed a perinuclear location of ADSC-EVs and their accumulation in vesicular-like structures within the SC cytoplasm. Upon comparing intracellular localization patterns of silica beads and ADSC-EVs in SCs, we found striking resemblance in size and distribution. Live cell imaging visualized that the uptake of ADSC-EVs preferentially took place at the SC processes from which the EVs were transported towards the nucleus. This study provided first evidence for an endocytosis mediated internalization of ADSC-EVs by SCs and underlines the therapeutic potential of ADSC-EVs in future approaches for nerve regeneration.

## 1. Introduction

Peripheral nerve injuries lead to motor and sensory deficits that still represent a therapeutic challenge with unsatisfactory outcome [1]. Remarkably, the peripheral nervous system (PNS) is capable to regenerate after minor injuries such as segmental demyelination (neurapraxia) or the disruption of axons when most of the surrounding connective tissue remains intact (axonotmesis) [2]. Severe injuries include nerve transection (neurotmesis) or the loss of nervous tissue that exceeds the PNS’ inherent regenerative ability. Such conditions commonly entail scar tissue infiltration and/or neuroma formation causing pain and permanent deficits that severely affect the patient’s life [3]. In addition, injury sites far from the target organ require axons to regenerate over long distances. As the mean growth rate of axons is 1 mm per day, the distal part may experience long-term denervation, which is associated with poor functional recovery [4,5].

The gold standard for the treatment of severe nerve injuries are microsurgical techniques [6,7]. In case of nerve transection, the nerve endings may be reconnected if secure and tension-free end-to-end nerve coaptation is possible [8]. Artificial nerve conduits are used to bridge nerve defects of about 4–8 mm and the transplantation of an autologous nerve graft is the current standard treatment to replace lost nerve tissue exceeding 8 mm [1,6,9]. Nerve autografts contain an intact nervous architecture, cellular support, and extracellular matrix (ECM) molecules that offer the best conditions possible for nerve recovery. Nevertheless, the availability of donor nerves is limited and donor nerve innervated tissue suffers from functional loss. The second incision site bears the additional risk of donor site morbidity, neuroma formation, and pain [7,10]. To overcome these disadvantages, current research focusses on the improvement of artificial nerve conduits or decellularized nerve allografts enriched with cells or biological components such as growth factors, peptides, or ECM molecules [11,12,13,14,15,16].

The first choice for cellular enrichment of nerve conduits, allografts, or chronically denervated nerves are Schwann cells (SCs). The regenerative potential of the PNS is attributed to their highly plastic cell state, which allows SCs to transform into a specialized repair cell after peripheral nerve injury [17]. Repair SCs create a regenerative environment and regulate the multistep process of nerve regeneration by conducting essential tasks that involve the phagocytic removal of myelin debris, the attraction of phagocytes, and neurotrophic support for injured axons [18,19,20,21]. In addition, repair SCs align in cords of cells (Bands of Büngner) to form a guiding structure that directs the regrowing axons towards their target [21]. The first autologous SC transplantation studies in humans showed promising results and demonstrated safety and clinical feasibility [22]. However, the clinical use of SCs is hampered because their harvest entails the sacrifice of healthy nerve tissue with associated side effects as well as challenges in their in vitro culture.

In the search for alternatives, mesenchymal stem cells, including adipose tissue-derived stem cells (ADSCs), turned into the focus of regenerative therapies [23]. ADSCs can be easily harvested, rapidly expanded in vitro, and secrete a variety of neurotrophic factors, cytokines and chemokines [24,25]. In line with these findings, several studies demonstrated a beneficial impact on nerve regeneration after introducing ADSCs [26,27,28]. Of note, there is increasing evidence that the regenerative effect of stem cells is also mediated via extracellular vesicles (EVs) [29,30,31,32,33]. EVs are defined as heterogenous entities of phospholipid bilayer delimited vesicles without any means of replication that differ in their biogenesis, composition and size [34,35,36]. To prevent further misuse and confusion about the terminology of EVs, broadly comprising the formerly known exosomes, micro-vesicles and apoptotic bodies, the Minimal Information for Studies of Extracellular Vesicles (MISEV) guidelines were established [34]. EVs are encouraged to be categorized according to their different properties such as physical characteristics (size and density), biochemical composition (marker expression), and the cell type and condition they have been obtained from [34]. EVs are released by numerous cells and act as paracrine and/or autocrine signaling factors [36,37]. The cargo of EVs consists of bioactive materials able to influence cellular behavior such as proteins, nucleic acids, and lipids located either on their membrane surfaces or within their luminal space [38,39,40,41]. Interaction of EVs with a recipient cell can occur by receptor binding, active and passive plasma membrane fusion, or endocytosis [37,42]. In addition, EVs may release their content into the extracellular space or act as antigen presenters [35,40]. Hence, ADSC derived EVs (ADSC-EVs) could offer a therapeutic alternative for regenerative approaches by avoiding the risk of cell-based therapies [29,30,31,43].

Recent studies showed that ADSC-EVs can facilitate peripheral nerve regeneration, presumably by supporting SC proliferation and migration [44,45]. This potential of EVs is of particular interest because the support and number of repair SCs was shown to progressively decline in chronically denervated nerves [4,5,46,47]. However, it was not yet investigated whether ADSC-EVs exert their beneficial effect on SCs via extracellular receptor binding, fusion to the SC membrane, or endocytosis mediated internalization. To accurately validate the proliferation rate of SCs in response to ADSC-EV treatment, a proliferation assay combined with a multicolor immunofluorescence staining panel and automated image analysis was established. In addition, we used confocal microscopy, high resolution 3-D analysis and live cell imaging to shed light on the uptake and localization of ADSC-EVs during/after co-culture with SCs.

## 2. Methods

### 2.1. Animals

For this study, sciatic nerve tissue was harvested from adult Sprague–Dawley rats for SC isolation. According to the Austrian’s Animal Testing Law (TVG 2012, §2, 1.c) and according to Article 3 of the *Directive 2010/63/EU of The European Parliament and of the Council on the Protection of Animals Used for Scientific Purposes* the sole tissue harvest from sacrificed/euthanized animals does not require an ethical approval.

### 2.2. Isolation and Culture of Primary Rat Schwann Cells

SCs were isolated, cultured, and enriched as previously described [20,48]. Briefly, sciatic nerves were digested and cultured in 0.01% poly-L-lysine hydrobromide (PLL, Sigma-Aldrich, St. Louis, MO, USA) and 5 µg/mL laminin (Sigma-Aldrich) coated dishes with Schwann cell culture medium consisting of MEMα (GlutaMAXTM-I, GIBCO, Waltham, MA, USA) supplemented with 2.5% HEPES (GIBCO), 1% penicillin-streptomycin (P/S, GIBCO), 1% sodium pyruvate (GIBCO), 5% (FCS, LINARIS, Dossenheim, Germany), 10 ng/mL recombinant heregulinβ-1 (PeproTech, London, UK), 0.5% N-2 supplement (GIBCO), 2 µM forskolin (Sigma-Aldrich), 10 ng/mL recombinant FGF basic (PeproTech), and 5 ng/mL PDGFAA (PeproTech). rSC cultures from passage 2 (p2) but not higher than p5 were used for experimentation. For the immunofluorescence staining analysis, 1 × 10^4^ rSCs were seeded per PLL/laminin-coated 8-well (μ-slides, Ibidi, Gräfelfing, Germany) in Schwann cell culture medium and grown until desired confluency. For the proliferation assay, 8 × 10^3^ rSCs were seeded per coated 8-well.

### 2.3. Isolation, Culture and, Differentiation of Primary Rat Adipose Stem Cells

Subcutaneous fat tissue was harvested, transferred to a falcon tube with fresh 1× PBS containing 1% antibiotic–antimycotic and further processed within 30 min after excision under sterile conditions. The fat tissue was manually cut into smaller pieces and then incubated with 1 mg/mL collagenase type CLS (type-1, Merck, Darmstadt, Germany) under shaking conditions for 1 h at 37 °C. The cell suspension was further dissociated by repeated pipetting, filtered through a 70 µm nylon cell strainer (FALCON, Corning Inc., Corning, NY, USA) and centrifuged at 300× *g* for 7 min. The pellet was resuspended and seeded in a T75 flask containing rADSC culture medium composed of DMEM high glucose (GIBCO) supplemented with 1% P/S, 10% FCS, 1% sodium pyruvate and 2 ng/mL recombinant FGF basic. The medium was changed every other day until the culture reached about 80% confluency. Then, cells were sub-cultured and seeded with a density of 3 × 10^4^ /cm^2^. For the immunofluorescence staining analysis of grown rADSCs in p1 and p3, 4 × 10^3^ cells were seeded per 8-well containing rADSC culture medium and grown until ~70% confluency. Multi-lineage differentiation potential of rADSCs at p3 was tested by adding adipogenic, chondrogenic, and osteogenic differentiation medium (PromoCell, Heidelberg, Germany) according to the manufacturer’s protocol.

### 2.4. Isolation of Rat Adipose Stem Cells-Derived Extracellular Vesicles

EVs were isolated from rADSC cultures in p3. When rADSC cultures reached about 80% confluency, the cells were washed three times with 1× PBS and incubated with culture medium without FCS for 12 h. The conditioned culture medium was centrifuged at 2000× *g* for 30 min at 4 °C. Isolation of rADSC-EVs from the supernatant was performed using the Total Exosome Isolation Reagent from cell culture medium (Invitrogen, Waltham, MA, USA). The rADSC-EV protein concentration was determined with the protein quantification assay (Macherey-Nagel, Düren, Germany). Aliquots of rADSC-EVs were stored in 1× PBS at −80 °C to avoid repeated freeze–thaw cycles.

### 2.5. Nanoparticle Tracking Analysis

The size distribution of isolated rADSC-EVs was quantified by nanoparticle tracking analysis (NTA) using the ZetaView device (ParticleMetrix, Meerbusch, Germany). Quality control of appropriate size distribution detection was performed using silica beads (1:250,000) provided by the manufacturer. A fixed measurement protocol was established for quantification of samples at 23 °C. The measurement mode included three cycles with 11 camera positions to detect particles of a maximal area of 5000 and minimal area of 6. Brightness and sensitivity were 60 and 65, respectively, and channels without detected particles were excluded manually. Measurements were repeated in technical replicates and the data was pooled for statistical analysis.

### 2.6. Immunofluorescence Staining

All antibody details are listed in Supplementary Table S1. The procedure was carried out at room temperature unless otherwise noted. The washing step involved a sequential incubation with 1× PBS for 5 min each. Grown cells were washed and fixed with 4.5% formaldehyde solution (SAV Liquid Production GmbH, Flintsbach am Inn, Germany) for 15 min. In case of extracellular protein staining, blocking was performed with 1× PBS containing 1% bovine serum albumin (BSA, Sigma-Aldrich) and 3% goat serum (DAKO, Agilent, Santa Clara, CA, USA) for 20 min. Subsequently, the cells were incubated with primary antibodies in 1× PBS containing 1% BSA and 1% goat serum overnight at 4 °C, washed, and incubated with respective secondary antibodies for 1 h. For intracellular protein staining, cells were blocked and permeabilized with 1× PBS containing 1% BSA, 0.3% TritonX–100 (Sigma-Aldrich), and 5% goat serum for 10 min. If required, the EdU detection was performed after permeabilization, see section proliferation assay. Then, the cells were incubated with primary antibodies in 1× PBS containing 1% BSA, 0.1% TritonX–100, and 1% goat serum for 2 h, washed, and incubated with respective secondary antibodies for 1 h. For nuclear staining, 50 µg/mL 4,6-Diamidino-2-Phenylindole solution (DAPI, ThermoScientific, Waltham, MA, USA) was added for 10 min. After washing, the cells were embedded in Fluoromount-G mounting medium (Invitrogen). The stained cells were stored at 4 °C for up to 2 weeks.

### 2.7. Western Blot

All antibody details are listed in Supplementary Table S1. The procedure was carried out at RT unless otherwise noted. 1× TBS-T was used for all washing steps that were performed three times for 5 min after each antibody incubation. Briefly, rADSC and rADSC-EV pellets were resuspended in RIPA buffer, sonicated for 15 min and mixed with SDS-loading buffer (Laemli buffer, BioRad, Hercules, CA, USA). Subsequently, samples were denatured for 5 min at 99 °C, separated on a 12% SDS-PAGE and blotted onto PVDF membranes (BioRad). Membranes were blocked using 1× TBS-T with 5% BSA for 1.5 h and incubated with primary antibodies in 1× TBS–T with 5% BSA overnight at 4 °C. After washing, respective secondary antibodies were added for 1 h at RT. The blots were analyzed with the Odyssey^®^ CL × Imaging System (Li–Cor Biosciences, Lincoln, NE, USA) and Image Studio software (V5.2).

### 2.8. Reverse Transcriptase Quantitative Polymerase Chain Reaction

RNA isolation of rADSCs and rADSC-EVs was performed with the RNeasy kit (Qiagen, Hilden, Germany). Subsequently, RNA concentration was measured (Nanophotometer, Implen, Munich, Germany) and 50 ng RNA was used for cDNA synthetization (iScriptTM cDNA Synthesis Kit, Biorad). The RT-qPCR was performed using SYBR^®^ Green Supermix (BioRad) and the 7500 Fast Real-Time PCR System (Applied Biosystems, Foster City, CA, USA); respective primer sequences are listed in Supplementary Table S2.

### 2.9. Fluorescence Labelling of Extracellular Vesicles

Isolated rADSC-EVs were slowly thawed on ice and fluorescently labelled using the PKH67 Green Fluorescent Cell Linker Mini Kit for General Cell Membrane Labeling (Sigma-Aldrich). The labelled rADSC-EVs were resuspended in Schwann cell culture medium and added to rSC cultures within 30 min.

### 2.10. Co-Culture of Rat Schwann Cells with Rat Adipose Stem Cells Derived Extracellular Vesicles

For the co-culture of rSCs with rADSC-EVs, 8 × 10^3^ rSCs were seeded per PLL/Laminin-coated 8-well with 250 µL rSC culture medium. The proliferation experiment was started as soon as the culture reached 40% confluency within 24 to 48 h. Then, 2 µg and 8 µg rADSC-EVs were added to the rSCs and incubated for either 24 h and 72 h, respectively (n = 3). Alongside, rSC cultures without the addition of rADSC-EVs served as control (CTRL).

### 2.11. Proliferation Assay

The proliferative effect of EVs on SCs was assessed using the Click-iT™ Plus EdU Alexa Fluor™ 555 Imaging Kit (Invitrogen). This proliferation assay is based on the incorporation of the thymidine analogue 5-ethynyl-2′-deoxyuridine (EdU) into DNA during the S-phase of the cell cycle. By adding a fluorescent azide the incorporated EdU can be detected via covalent cross-linking (click–reaction). This enables the visualization of newly synthesized DNA within a cell. After 24 or 72 h of rADSC-EV addition, 10 μM EdU was added to the rSC cultures and incubated for 2 h. Afterwards, the cells were fixed using 4.5% formaldehyde solution for 15 min at room temperature. For EdU detection the cells were permeabilized with 0.3% Triton X-100 for 10 min and the following steps were performed according to the manufacturer’s protocol. Subsequently, the immunofluorescence staining was performed; see section immunofluorescence staining.

### 2.12. Confocal Fluorescence Microscopy

Immunofluorescence images were taken using a SP8X confocal laser scanning fluorescence microscope (Leica Microsystems GmbH, Wetzlar, Germany). Additionally, a LSM 780 confocal laser scanning fluorescence microscope (Carl Zeiss Microscopy GmbH, Jena, Germany) equipped with Airyscan technology was used for 3-D analysis of rADSC-EVs. The Airyscan detector combines 32 detector elements that act as individual pinholes. Images from all 32 detector elements are combined by linear deconvolution, resulting in an increased signal-to-noise ratio and a 1.7× increase in resolution [49]. To further enhance the resolution of PKH67-labelled rADSC-EVs images, they were processed with Huygens Pro Deconvolution Wizard (Scientific Volume Imaging, Hilversum, The Netherlands). Images are depicted as maximum intensity projections of total z-stacks and artificial coloring was applied to make multicolor figures comprehensible for color-blind readers.

### 2.13. Analysis Software

To analyze rSC proliferation, the CellProfiler V3.1.8 open source software [50] and the Fiji open source platform [51] were used. Initially, .tif-files of single channel images were converted within Fiji to greyscale 8-bit .tif-files using the BatchConverter plugin. A customized CellProfiler pipeline was established to extract information about SC identity and proliferation status from SOX10, EdU, and DAPI stained immunofluorescence images. In this way, SOX10^+^/DAPI^+^ rSCs and SOX10^+^/EdU^+^/DAPI^+^ proliferating rSCs were automatically quantified and exported into a spreadsheet.

### 2.14. Statistical Analysis

The data were statistically analyzed with GraphPad Prism 6. The results of neurotrophic factor expression between rADSCs and corresponding rADSC-EVs are given as mean ± standard deviation (n = 5), a paired t-test was used to calculate significant differences. The results of rSC purity and proliferation in response to two different doses of rADSC-EV treatment compared to untreated controls are given as mean ± standard deviation (n = 3); a one way ANOVA (analysis of variance) with Tukey’s post hoc test was performed for multiple comparison test. A *p*-value <0.05 was considered significant.

## 3. Results

### 3.1. Characterization of rADSCs and rADSC-EVs

**Figure 1:**
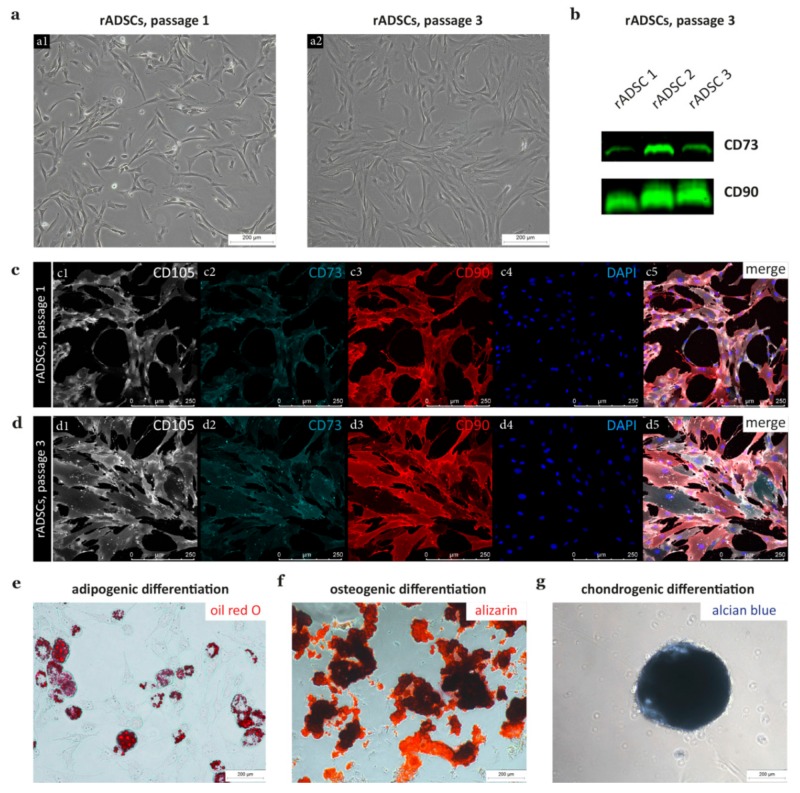
Characterization of rat adipose tissue derived stem cells (rADSCs). (**a**) Phase contrast images of rADSCs in p1 (**a1**) and p3 (**a2**). (**b**) Western blot results for three rADSC donors double stained against CD73 and CD90; whole blots are depicted in Supplementary Figure S1a. Immunofluorescence staining of p1 (**c**) and p3 (**d**) rADSC cultures for CD105 (**c1**,**d1**), CD73 (**c2**,**d2**), CD90 (**c3**,**d3**), DAPI (**c4**,**d4**), and merged channels (**c5**,**d5**). Induction of adipogenic differentiation shown by the appearance of fat droplets positive for oil red O staining (**e**), osteogenic differentiation by positive alizarin red staining (**f**), and chondrogenic differentiation by typical spheroid formation positive for alcian blue (**g**).

Cell outgrowth of p0 rADSCs was observed about two days after seeding. rADSCs in p1 showed an elongated morphology that changed to a more broadened appearance with increased culture time (Figure 1(a1,a2)). The expression of stem-cell-associated marker combinations was demonstrated by Western Blot for CD73 and CD90 (Figure 1b) and by multicolor immunofluorescence stainings including CD105, CD73, and CD90 (Figure 1c,d). We validated all used antibodies in single stainings and excluded unspecific staining signals by the presence of positive and negative cell populations for CD105, CD73, and CD90 (Supplementary Figure S1b–d). Compared to p1 rADSC cultures, we found an increased expression of CD73 in p3 rADSCs (Figure 1(c2) versus Figure 1(d2)). The lack of immune and hematopoietic cells was confirmed by negative stainings for CD11b, CD45, and CD34 (Supplementary Figure S1d–f). In addition, the multi-lineage differentiation potential of rADSCs was successfully demonstrated for adipogenic (Figure 1e), osteogenic (Figure 1f), and chondrogenic (Figure 1g) differentiation.

**Figure 2:**
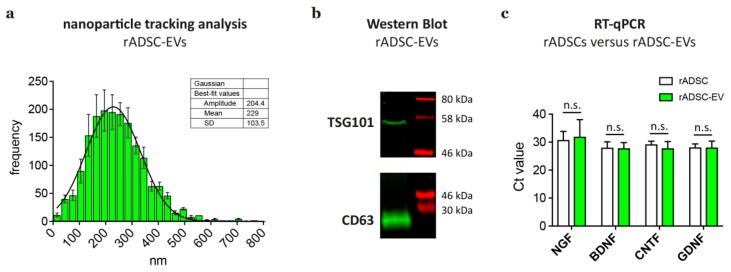
Characterization of rat adipose tissue derived stem cell derived extracellular vesicles (rADSCs-EVs). (**a**) rADSC-EV size distribution results determined by nanoparticle tracking analysis. (**b**) Western blot results of pooled batches of rADSC-EVs stained against TSG101 and CD63; whole blots are depicted in Supplementary Figure S1h. (**c**) RT-qPCR results for RNA expression levels of growth factors *NGF*, *BDNF*, *CNTF*, and *GDNF* in rADSCs and corresponding rADSC-EVs derived from 5 donors. Data are depicted as mean + SD (n = 5); not significant (n.s.).

Isolated rADSC-EVs were characterized in accordance to the MISEV 2018 guidelines regarding size and biochemical composition [34]. Nanoparticle tracking analysis detected a size distribution of rADSC-EVs from about 30 to 500 nm, with a mean particle size of 229 nm (Figure 2a). Western blot analysis confirmed the expression of EV-associated markers TSG101 and CD63 in pooled fractions of isolated rADSC-EVs (Figure 2b). Previous studies further showed that ADSCs and ADSC-EVs express neurotrophic factors [24,44]. Therefore, we compared the expression of the neurotrophic factors *NGF*, *BDNF*, *CNTF*, and *GDNF* between rADSCs and rADSC-EVs as an additional characterization step. The results confirmed that both rADSCs and rADSC-EVs contained mRNAs of *NGF*, *BDNF*, *CNTF*, and *GDNF* but showed no significant differences in their expression levels (Figure 2c).

### 3.2. Characterisation of rSCs

**Figure 3:**
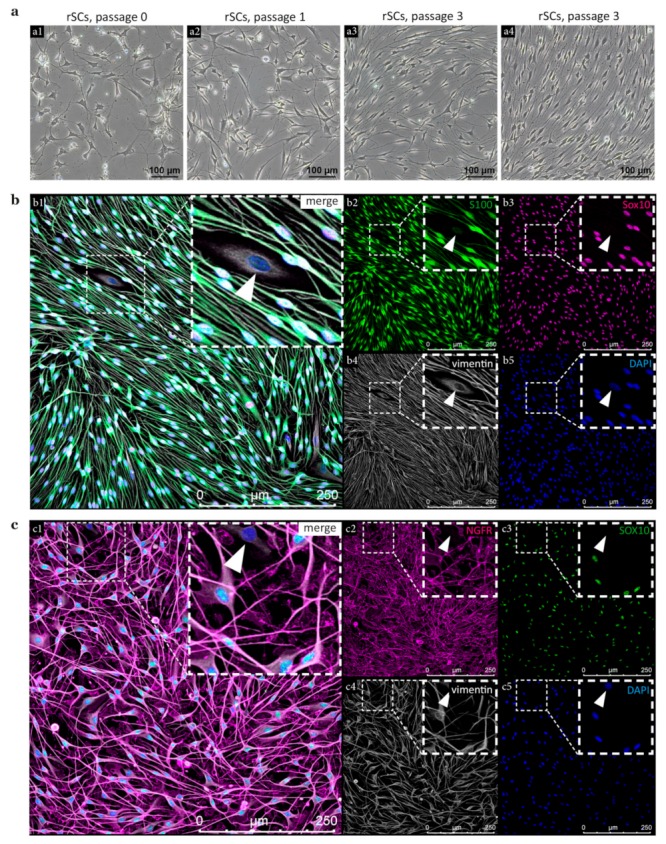
Characterisation of rSCs. (**a**) Phase contrast images of rSCs in p0 (**a1**), p1 (**a2**), and p3 (**a3**,**a4**). (**b**) Immunofluorescence staining of p3 rSC cultures showing merged staining channels (**b1**) and single staining channels for S100 (**b2**), SOX10 (**b3**), vimentin (**b4**), DAPI (**b5**); arrowheads indicate a SOX10^−^ FB with weak S100^+^ signals in the nucleus. (**c**) Immunofluorescence staining of p3 rSC cultures showing merged staining channels (**c1**) and single staining channels for NGFR (**c2**), SOX10 (**c3**), vimentin (**c4**), DAPI (**c5**); arrowheads indicate a NGFR^−^/SOX10^−^ FB.

The morphology of rSC cultures was monitored over several passages (Figure 3a). Cultures in p0 contained fibroblasts (rFBs) with a broad and flattened morphology and rSCs characterized by their long, bi- to multi-polar processes (Figure 3(a1)). During passaging, the rFBs were depleted by exploiting the differential adhesion properties of rSCs and rFBs. With increased culture time, rSCs adapted the typical spindle-shaped morphology and showed an increased parallel alignment (Figure 3(a2–a4)). Multicolor immunofluorescence stainings confirmed the expression of the SC-associated calcium binding protein S100 and glial transcription factor SOX10 in rSCs (Figure 3b). Notably, some rFBs showed weak signals of S100 in the nucleus (Figure 3b, arrowheads), while SOX10 was exclusively expressed by rSCs. Both rSCs and rFBs were positive for the intermediate filament vimentin, which also visualized the long SC processes (Figure 3b).

Furthermore, we tested the expression of SOX10 together with SC marker NGFR (low affinity nerve growth factor receptor, also known as p75) that showed a strong membranous staining on rSCs (Figure 3c). rFBs lacked the expression of both NGFR and SOX10 (Figure 3c, arrowheads), which encouraged their use as reliable SC markers in the following experiments.

### 3.3. rADSC-EVs Elevate the Proliferation of rSCs In Vitro

**Figure 4:**
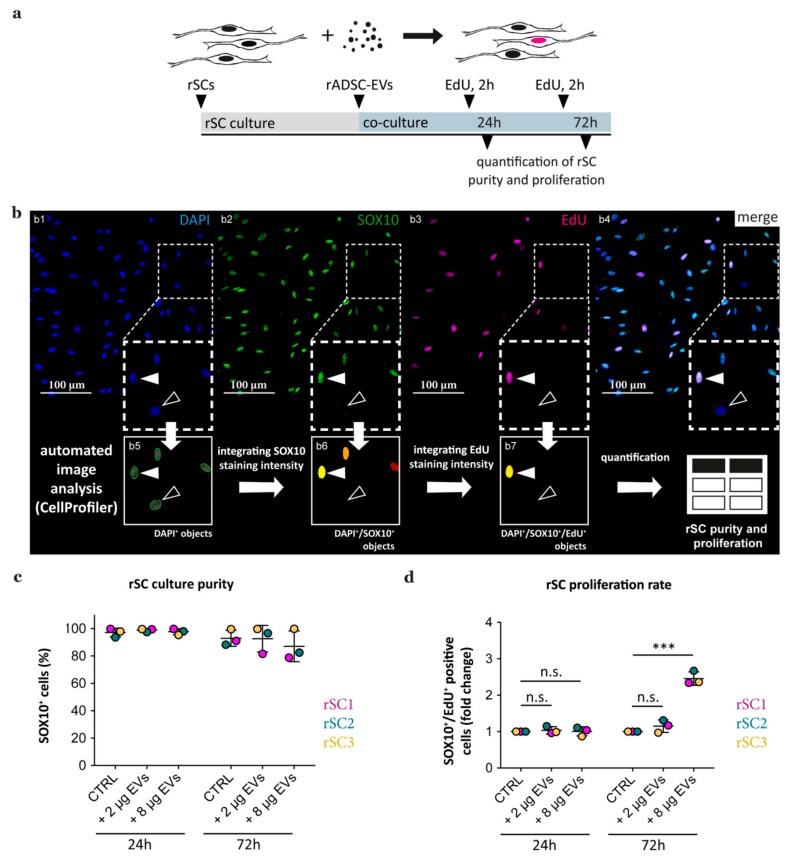
Quantification of rat Schwann cell (rSC) purity and proliferation in response to rADSC-EVs. (**a**) Set up of the proliferation experiment. (**b**) Immunofluorescence staining of rSCs stained for DAPI (**b1**), SOX10 (**b2**), EdU (**b3**), and merged channels (**b4**). Cell profiler analysis steps of respective enlargements showing DAPI^+^ object segmentation (**b5**) and the identification of DAPI^+^/SOX10^+^ cells (= SCs) (**b6**) and DAPI^+^/SOX10^+^/EdU^+^ cells (= SCs in S-Phase of the cell cycle) (**b7**); filled arrowheads indicate a DAPI^+^/SOX10^+^/Edu^+^ proliferating SC, lined arrowheads indicate a DAPI^+^/SOX10^−^/EdU^−^ FB. (**c**,**d**) Evaluation of the purity and proliferation rate of SC cultures derived from 3 donors (rSC1, rSC2, and rSC3) in response to rADSC-EV treatment. (**c**) rSC culture purities according to the calculated number of DAPI+/SOX10+ cells in controls (CTRL) and after treatment with 2 µg and 8 µg EVs for 24 h and 72 h; >600 cells were analysed per condition. (**d**) The proliferation rate of SCs treated with 2 µg and 8 µg rADSC-EVs normalized to CTRLs after 24 h and 72 h, respectively; >600 cells were analyzed per condition. Data are depicted as mean + SD (n = 3); *** *p*-value < 0.001, not significant (n.s.).

Next, we validated whether rADSC-EVs exert a proliferative effect on rSCs in vitro. Therefore, we treated rSCs with two different concentrations of rADSC-EVs and analyzed the proliferation status of rSCs by EdU incorporation after 24 h and 72 h of co-culture (Figure 4a). At the respective end point, treated and non-treated rSC cultures were stained for EdU, DAPI, as well as SC marker SOX10 to discriminate proliferating rSCs from rFBs (Figure 4(b1–b4), filled versus lined arrowheads, respectively). For the determination of SC culture purity and proliferation, we adapted an automated image analysis pipeline provided by the CellProfiler software. The main image analysis steps involved the segmentation of DAPI^+^ cell nuclei followed by the identification of DAPI^+^/SOX10^+^ objects (SCs) and DAPI^+^/SOX10^+^/EdU^+^ objects (proliferating SCs) (Figure 4(b5–b7)). The results showed that the rSCs cultured for 24 h had a purity of about 96% and that emerging numbers of FBs eventually lead to a reduced SC culture purity after 72 h (Figure 4c). The proliferation assay determined that no significant difference in rSC proliferation was detected after 24 h in both the 8 µg and 2 µg rADSC-EV treated groups (Figure 4d). However, the proliferation of rSCs treated with 8 µg rADSC-EVs was significantly increased about 2.5 fold after 72 h compared to untreated controls. This finding demonstrates that the proliferative effect of rADSC-EVs on rSCs is time- and dose-dependent (Figure 4d).

### 3.4. rADSC-EVs Are Internalized by SCs and Accumulate in Vesicular Structures

**Figure 5:**
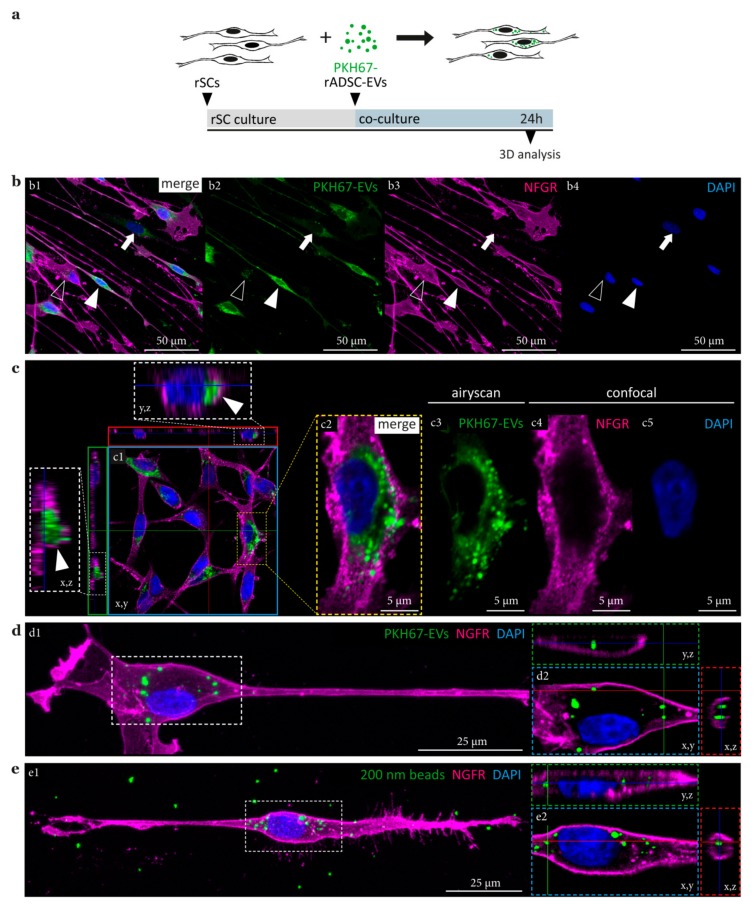
3-D analysis of rSCs upon treatment with rADSC-EVs and silica beads. (**a**) Set up of the co-culture experiment. (**b**,**c**) Immunofluorescence images of rSCs co-cultured with rADSC-EVs for 24 h; merged channels (**b1**,**c1**,**c2**) and single channels showing PKH67-labeled rADSC-EVs (**b2**,**c3**), NGFR (**b3**,**c4**) and DAPI (**b4**,**c5**) stainings. (**b**) Filled arrowheads indicate a rSC with intracellular staining signals of rADSC-EVs homogenously distributed within the SC body and processes; lined arrowheads indicate a rSC with rADSC-EV staining signals of a dotted appearance within the cell body; arrows indicate a rFB with rADSC-EV staining signals around the nucleus. (**c**) High resolution 3-D analysis of rADSC-EVs in rSCs. (**c1**) Image represents a selected focal plane of a z-stack; arrows in y,z and x,z cross sections indicate rADSC-EVs within the cytoplasm; (**c2**) Enlargement of (**c1**) showing the Airyscan result for PKH67-EVs (**c3**) as well as confocal images of NGFR (**c4**) and DAPI (**c5**) stainings. (**d**,**e**) Images of single rSCs after treatment with (**d1**) PKH67-labeled rADSC-EVs and (**e1**) green fluorescent 200 nm beads. Selected focal planes and cross sections of cell body enlargements for (**d2**) PKH67-labeled rADSC-EVs and (**e2**) 200 nm green fluorescent silica beads.

PKH dyes are commonly used for live cell labelling as their aliphatic tails anchor into the lipid bilayer of the plasma membrane. The biogenesis of EVs causes their delimitation by a lipid bilayer, which allows EV membrane labelling with PKH dyes [52]. The fate of PKH67-labeled ADSC-EVs upon co-culture with rSCs was analyzed by confocal microscopy (Figure 5a). After 24 h of co-culture, the rADSC-EVs appeared predominantly localized around the cell nuclei of both rSCs (Figure 5b, arrowheads) and rFBs (Figure 5b, arrows). Notably, we found that the amount and distribution of internalized rADSC-EVs differed between cells, including a dotted perinuclear appearance (Figure 5b, lined arrowheads) and a more homogenous distribution within the cell body and cellular processes (Figure 5b, full arrowheads). To confirm that the rADSC-EVs are not only bound to but indeed internalized by SCs, we took images at different focal planes with a distance of 0.2 µm to cover the entire cell volume. 3-D analysis revealed a cytoplasmic localization of rADSC-EVs, beneath the NGFR positive SC membrane (Figure 5(c1), arrows). Due to their small size, the resolution of EVs by conventional confocal microscope detectors is poor. Hence, Airyscanning was used to retrieve a 1.7× increase in resolution for the analysis of internalized rADSC-EVs. This way we could show that internalized rADSC-EVs signals accumulated in vesicular-like structures of different size (about 0.5–2 µm) within the SC cytoplasm (Figure 5(c2–c5)).

It was previously demonstrated that cells internalize silica beads with a size of about 50 and 300 nm via different endocytic pathways and store them in endosomal/lysosomal compartments [53,54]. To compare the fate of rADSC-EVs with that of silica beads, we challenged rSCs with green-fluorescent beads of 200 nm in size, thus, comparable to the average size of rADSC-EVs used in this study (see Figure 2a). The results showed a similar appearance, size, and subcellular location of vesicular structures containing rADSC-EVs or 200 nm beads, respectively (Figure 5d versus Figure 5e), which supports an endocytosis-mediated internalization pathway of rADSC-EVs.

### 3.5. Live Cell Imaging of rADSC-EVs/rSC Co-Cultures

**Figure 6:**
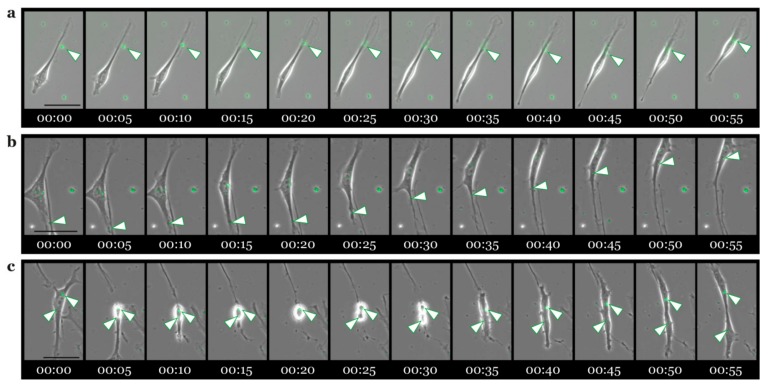
Live cell imaging of rSCs treated with rADSC-EVs. The Live cell imaging series in (**a**,**b**) visualize the binding and uptake of rADSC-EVs at the rSC processes followed by their transport to the cell body. The Live cell imaging series in (**c**) depict a dividing rSC with internalized rADSC-EVs. Scale bars in (**a**–**c**) represent 50 µm.

To provide information about the uptake of rADSC-EVs by rSCs, we monitored co-cultures by live cell imaging. Therefore, PKH67-labeled rADSC-EVs were added to rSC cultures and pictures were taken every 5 min for 24 h. Although PKH67-labeled rADSC-EVs with an average size of 200 nm are too small to be detected by conventional fluorescent microscopy, clusters of rADSC-EVs can be observed and followed. We found that the binding of rADSC-EV clusters preferentially took place at the lamellipodia of SC processes (Figure 6a). Subsequently, the internalized rADSC-EVs were transported towards the cell body (Figure 6a,b). In addition, we observed that vesicular-like structures of rADSC-EVs are distributed in the two daughter cells after cell division (Figure 6c).

## 4. Discussion

The regenerative potential of the PNS is largely dependent on the transformation of adult SCs into a dedicated repair cell. This process involves the re-expression of immature/precursor SC associated genes, proliferation, and the acquisition of repair specific functions. Repair SCs not only directly support the survival and guidance of axons but also govern a regenerative environment by shaping the ECM and facilitating an immunological response. However, when repair SCs experience prolonged lack of axonal contact, their regenerative functions diminish and cell numbers decline [4,5]. These events could be responsible for the poor functional recovery of chronically denervated nerves. The identification of factors sustaining the SCs’ repair phenotype is therefore of high therapeutic interest for chronic denervation injuries as well as the enrichment of artificial nerve conduits and decellularized nerve allografts.

Recent studies demonstrated that mesenchymal stem cell-derived EVs are able to promote regeneration of the nervous system by enhancing axonal outgrowth and promoting angiogenesis [33,55,56]. Accordingly, also ADSC-EVs have been shown to improve regeneration after nerve crush injury and a bridged 7-mm nerve gap in rats presumably by affecting SC proliferation, migration, and myelination [44,45]. Thus, understanding the underlying molecular mechanisms bears a considerable potential to unveil novel strategies for therapeutic approaches. In this study, we investigated the effect and fate of ADSCs-EVs after their co-culture with SCs in detail. Both ADSCs-EVs and SCs were obtained from rats (rADSC-EVs and rSCs) to avoid cross-species related artefacts and co-cultured for 24 and 72 h. As it is known that SC cultures also contain FBs that may grow in number with prolonged culture time, we put emphasis on verifying a SC identity of analyzed cells. By including the SC marker SOX10 in the proliferation staining panel, we were able to discriminate SOX10^+^ SCs from SOX10^−^ FBs and exclusively determine the proliferation status of SCs. We could confirm that rADSC-EVs increased the proliferation of rSCs after 72 h but not after 24 h of co-culture. We also found a varying purity of rSC cultures after 72 h which was due to an augmented rFB population. This finding underlined the importance to implement an SC marker in the proliferation assay and verify that an increase of EdU signals is caused by the EVs and not by proliferating FBs.

It was claimed that the effect of ADSC-EVs on SCs is mediated via internalization but these studies did not prove an intracellular location of EVs [44,45]. Indeed, EVs can influence cellular processes by different modes of actions. They may bind to membrane surface receptors causing an intracellular signaling cascade without the delivery of their content. EVs could also release their cargo into the cytoplasmatic space by fusion with the recipient cell’s plasma membrane or, upon internalization, by fusion with the endosomal membrane through yet unknown molecular mechanisms [36,37]. In addition, EVs may release their content into the extracellular space [35]. Hence, it is important to address these different possibilities and define the molecular mechanisms behind a possible therapeutic application. Therefore, we performed 3-D analysis and investigated the localization of PKH67-labeled rADSC-EVs after co-culture with rSCs for 24 h. Confocal microscopy was used to take images at different focal planes with a distance of 0.2 µm. This way, the whole rSC volume was covered, which enabled us to assess the distribution and localization of PKH67-EV staining signals. The results demonstrated that rADSC-EVs were internalized by rSCs and mainly localized around the nucleus beneath the NGFR-positive rSC membrane. The increased resolution achieved by Airyscanning further allowed assigning the rADSC-EVs signals to vesicular-like structures of about 0.5 to 2 µm in size. The lack of PKH67 signals within the SC membrane further strengthened an endocytic uptake as upon fusion, the PKH67-labeled membranous components of rADSC-EVs would have been distributed within the SC membrane. These findings suggest that rADSC-EVs enter the rSC via endocytosis-related processes and do not bind to or fuse with the rSC membrane.

We next compared the distribution, size, and localisation pattern of internalized rADSC-EVs with material of similar size shown to be taken up via endocytosis. Silica beads with a size of 50 and 300 nm were reported to be incorporated by cells via different endocytic pathways and stored in endosomal/lysosomal compartments [53,54]. Therefore, we added green-fluorescent silica beads with a size of 200 nm to rSCs for 24 h. Following 3-D analysis, we could confirm that the appearance, size, and subcellular location of silica beads strikingly resembled those of PKH67-labeled rADSC-EVs. During the analysis of rSCs treated with PKH67-labeled rADSC-EVs, we also found that some rSCs possessed very homogenously distributed PKH67 signals within the cell body and cellular processes. This observation could represent rADSC-EVs within small endocytic vesicles not yet fused into larger endosomal compartments. The live cell imaging data illustrated that rADSC-EV clusters are preferentially bound and internalized at the rSC processes followed by their transport towards the cell body. It is well established that newly formed vesicles entering the endocytic pathways are transported away from the actin-rich cell periphery to a microtubule based transport system [57,58]. The long processes of SCs may involve similar retrograde transport mechanisms as described for neurons, which could result in the accumulation of rADSC-EV-filled vesicular structures within the rSC body.

It remains to be evaluated how the internalized rADSC-EVs exert their proliferative effect on rSCs. Since our results indicate that rADSC-EVs do not fuse with the rSC plasma membrane but rather enter the cell via endocytosis-related mechanisms, rADSC-EVs presumably release their cargo via fusion with the endosomal membrane. Potential effector molecules transported by EVs are small non-coding RNAs (microRNAs), which are able to modulate gene expression by post-transcriptional mechanisms [59,60,61]. MicroRNAs were shown to play an important role in modulating the cellular behavior of neurons and SCs in response to injury [60,61,62,63]. The pro-regenerative effect of EVs derived from different sources after nerve injury is indeed associated with transported microRNAs, reviewed in [64]. In addition, it was recently demonstrated that ADSC-EVs contain mRNAs of neurotrophic factors [44] and we confirmed the presence of NGF, BDNF, CNTF, and GDNF in rADSC-EVs. Thus, the cargo of ADSC-EVs hold a valuable therapeutic potential to support the SC repair phenotype by delivering mRNAs as well as microRNAs that increase neurotrophic factor release and proliferation, but presumably also modulate other regenerative functions.

In conclusion, we confirmed that rADSC-EVs are able to promote the proliferation of rSCs in a time- and dose-dependent manner. This study also provides first evidence for the internalization of rADSC-EVs by rSCs. We here demonstrate that rADSCs do not fuse with the rSC plasma membrane but accumulate in vesicular-like structures around the cell nucleus, which suggests an endocytosis-mediated internalization pathway. Further investigation is required to characterize the kind of endocytic pathway and to define the cargo of rADSC-EVs responsible for the proliferative effect on rSCs. Exploiting these molecules is promising to support the repair SC phenotype in therapeutic approaches for nerve regeneration.

## Supplementary Materials

The following are available online at https://www.mdpi.com/2073-4409/9/1/163/s1, Figure S1: Characterisation of rADSCs and rADSC-EVs., Table S1: Antibodies, Table S2: Primer sequences used for RT-PCR.
