# Supplementary material for: Adipose Stem Cell-Derived Extracellular Vesicles Induce Proliferation of Schwann Cells via Internalization
Source: Cells. 2020 Jan 9;9(1):163. doi: 10.3390/cells9010163 (PMC7016740; doi:10.3390/cells9010163)
Supplement: Supplementary file 1 [file cells-09-00163-s001.pdf]

SUPPLEMENTARY FIGURE

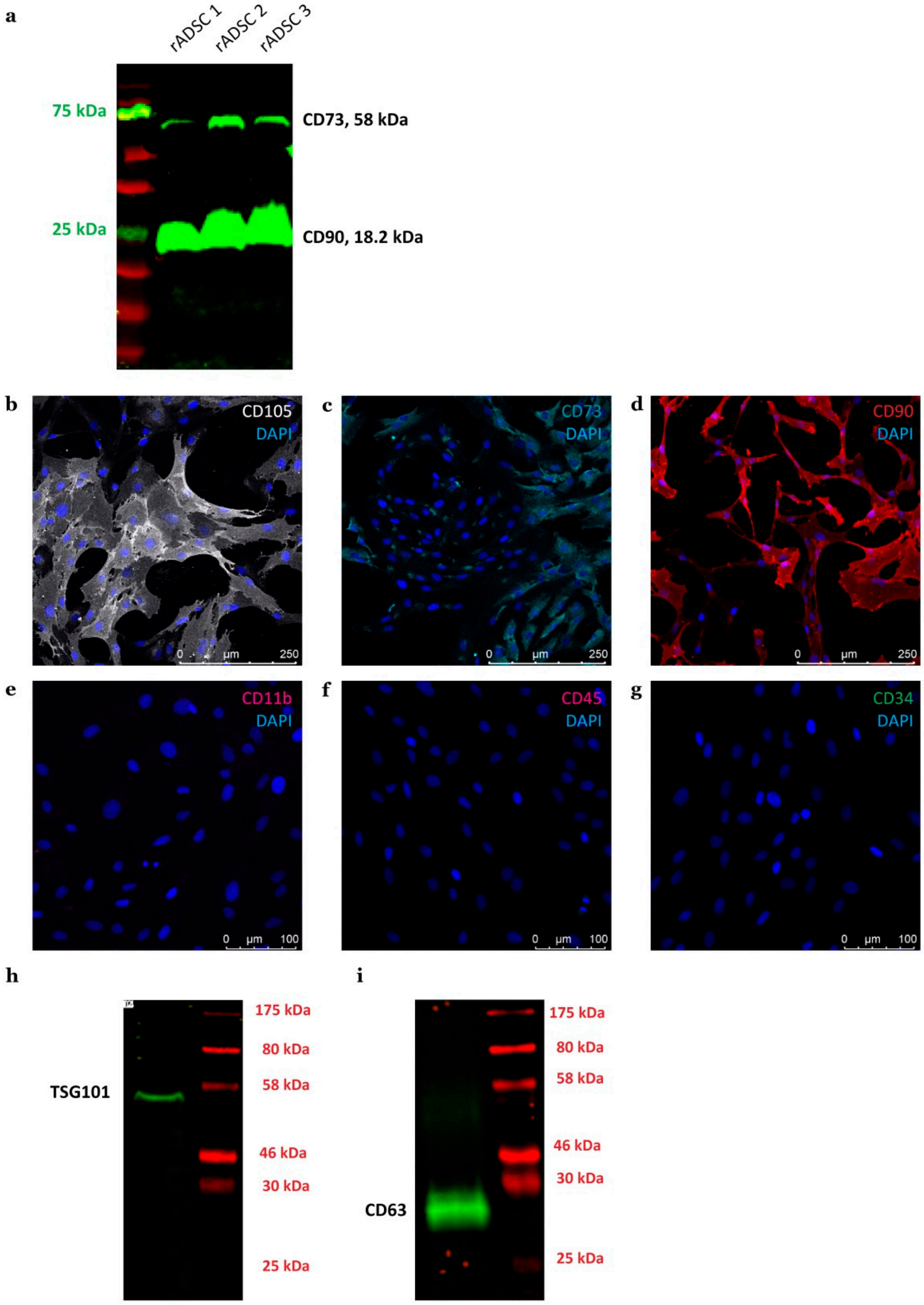

Supplementary figure 1. Characterisation of rADSCs. and rADSC-EVs. Whole Western blot for three rADSC donors double stained against CD73 and CD90 **(a)**. Immunofluorescence images of p3 rADSC cultures stained for CD105 **(b)**, CD73 **(c)** and CD90 **(d)**, note that positive cells can be clearly discriminated from negative cells. Immunofluorescence analysis of p3 rADSC cultures stained for CD11b **(e)**, CD45 **(f)** and CD34 **(g)**; note that no positive cells were observed. Whole Western blots for pooled rADSC-EV fractions stained against TSG101 **(h)** and CD63 **(i)**.

SUPPLEMENTARY TABELS

Supplementary Table 1 | Antibodies

1st Antibodies				
Reactivity	Species	company	dilution	note
S100	rabbit	DAKO	1:200	permeabilization
SOX10	mouse	Santa Cruz	1:50	permeabilization
vimentin	chicken	Invitrogen	1:200	permeabilization
NGFR	rabbit	CellSignaling	1:400	
CD90	mouse	Santa Cruz	1:50	
CD73	rabbit	CellSignaling	1:50	
CD105	goat	R&D	1:100	
CD11b	rat	ThermoFisher	1:100	
CD45	rabbit	Santa Cruz	1:30	
CD34	mouse	Santa Cruz	1:30	
TSG101	rabbit	abcam	1:500	
CD63	mouse	abcam	1:500	

2nd Antibodies			
Reactivity	Fluorophore	company	dilution
rabbit	AF488Plus	Invitrogen	1:600
mouse	AF594	Invitrogen	1:300
mouse	AF488	Invitrogen	1:300
chicken	DL650	Invitrogen	1:400
goat	AF546	Invitrogen	1:300
rat	AF594	Invitrogen	1:400
rabbit	IRDye 800CW	LI-COR	1:15000
mouse	IRDye 800CW	LI-COR	1:15000

Supplementary Table 2 | Primer sequences used for RT-PCR

Gene	Primer sequence
NGF	Foward: ATCGCTCTCCTTCACAGAGTTT
	Reverse: TGTACGGTTCTGCCTGTACG
BDNF	Foward: TACCTGGATGCCGCAAACAT
	Reverse: TGGCCTTTTGATACCGGGAC
CNTF	Foward: ATGGCTTTCGCAGAGCAAAC
	Reverse: CAACGATCAGTGCTTGCCAC
GDNF	Foward: CGCTGACCAGTGACTCCAAT
	Reverse: TGGTAAACCAGGCTGTCGTC
